# Corncob-Derived Activated Carbon as Electrode Material for High-Performance Supercapacitor

**DOI:** 10.3390/ma17174341

**Published:** 2024-09-02

**Authors:** Lili Dong, Chenghao Pan, Yongfeng Ji, Suxia Ren, Tingzhou Lei

**Affiliations:** 1 Institute of Urban & Rural Mining, Changzhou University, Changzhou 213164, Chinarensuxia@cczu.edu.cn (S.R.); 2 Changzhou Key Laboratory of Biomass Green, Safe & High Value Utilization Technology, Changzhou 213164, China

**Keywords:** supercapacitors, biomass, corncob, activated carbon, specific capacitance

## Abstract

In this study, corncob was explored as a low-cost and abundant precursor for the preparation of activated carbon via carbonization and the KOH activation method. The alkaline/biochar ratios varied from 3:1 to 5:1, and the activation temperatures ranged from 700 to 900 °C. The characterized results reveal that the alkaline/biochar ratios and activation temperatures had a remarkable influence on the morphology and microstructure of as-prepared activated carbon (CAC_T-R_). The CAC_T-R_ presented a porous structure with a large number of micropores and a small number of mesopores. The reasonable distribution of micropores and mesopores endows the ideal structure for ion transfer and charge storage. The optimal sample CAC_700-4_ exhibited the best capacitive performance with a specific capacitance of 260 F/g at 1 A/g. Moreover, the assembled CAC_700-4_//CAC_700-4_ symmetric supercapacitor showed a high energy density of 14.3 Wh/kg at a power density of 250 W/kg in 6 M KOH electrolyte. It also has a capacitance retention of 95.5% after 10,000 cycles, indicating its excellent cycle stability. These results indicate that corncob-derived activated carbon provides the possible application of biomass waste in high-performance supercapacitors.

## 1. Introduction

Supercapacitors with high energy density, fast charging and discharging rates, and excellent security present huge application potential in hybrid electrical vehicles and electronic devices [1,2]. It is known that electrode materials are the key factors affecting supercapacitor performance [3,4,5,6]. In recent years, numerous forms of carbon materials, including activated carbon, carbon aerogel, graphene, and other nanostructured carbon materials, have been explored as supercapacitor electrodes [7,8,9,10]. Nevertheless, graphene, carbon nanofibers, and carbon nanotubes tend to be used for the fabrication of hybrid electrodes, whereas activated carbon is identified as a qualified supercapacitor electrode material due to its reasonable porous structure, high surface area, stable performance, and abundant raw materials. Unfortunately, activated carbons are usually prepared from fossil materials, which are expensive and nonrenewable. Therefore, developing alternative low-cost and renewable precursors has attracted considerable attention.

In recent years, biomass wastes have been identified as promising precursors to activated carbon due to their unique porous structure and uniform distribution of heteroatoms [11,12,13,14]. Jin et al. used daylily as the carbon source to prepare heteroatom-rich carbon materials [15]. The resulting carbon material exhibited a three-dimensional porous structure with a specific capacitance of 299.1 F/g at 0.5 A/g. Wang et al. used camellia oleifera branches as a precursor to prepare porous carbon material, in which NH_4_Cl was used as a N dopant and activator agent. The optimal sample presented a hierarchical porous structure, which contributes to obtaining high specific capacitance [16]. Arkhipova et al. used rice husk as the precursor to prepare activated carbon through the KOH activation and carbonization method. The prepared samples presented a high specific capacitance of 400 F/g at 0.5 A/g, which were measured in 1 M Na_2_SO_4_ and 0.03 M K_3_Fe[CN]_6_ electrolyte [17]. Among various waste biomass, corncob has been identified as a major agricultural biomass waste in the world, and the productivity in China is up to 20 million tons per year. Corncob might be a promising raw material for high-performance carbon electrodes due to its nature of abundance, inhomogeneity, and low cost.

Based on the double-layer capacitance mechanism, high surface area and reasonable pore size distribution are the key factors to gain high capacitive performance [18,19]. Generally speaking, carbon materials for supercapacitors should possess a porous structure with a large amount of micropores, adequate mesopores, and some macropores. The numerous micropores mainly contribute to the large surface area to obtain a high double-layer capacitance. The mesopores mainly guarantee the transfer and diffusion of electrolyte ions, whereas the macropores are used to reduce the transport distance of electrolyte-to-electrode. Hence, numerous efforts have been focused on fabricating hierarchical porous carbons which meet the requirements. Among various pore-making methods, high-temperature calcination of carbon precursor mixed with KOH activator has been identified as the most effective method. Thus, the adjustment of the KOH activation parameter is crucial to obtaining high-performance activated carbons for supercapacitors.

In this paper, corncob-derived activated carbon was synthesized through carbonization and the KOH activation method. The effects of activation temperature and KOH/biochar ratio on the morphology, surface area, and microstructure of corncob-based activated carbon were studied in detail. The capacitive performance of the activated carbon was further analyzed using cyclic voltammetry and constant current charging and discharging methods.

## 2. Materials and Methods

### 2.1. Materials and Chemicals

The corncobs were from the suburb of Luoyang, Henan Province. Hydrochloric acid (HCl) and potassium hydroxide (KOH) were bought from Sinopharm Chemical Reagent Co., Ltd. (Shanghai, China). Polytetrafluoroethylene and acetylene black were provided by Shenzhen Kejing Technology Co., Ltd. (Shenzhen, China). All reagents were of an analytical grade and used as received.

### 2.2. Preparation of CAC_x_

Corncobs were first crushed to less than 1 mm. Next, 100 g of corncob powder was placed in a nickel crucible, which was then encapsulated with calcined coke. The above corncob was heated at 550 °C for 1 h to obtain biochar (CB). Then, 20 g of the resulting CB was mixed with KOH for 30 min in a three-dimensional mixer, with a mass ratio of 1:R (R = 3, 4, 5). The mixture was placed in a nickel crucible and encapsulated with calcined coke. The samples were first heated at 500 °C for 2 h in a Si Mo furnace and then at T °C (T = 700, 800, and 900 °C) for 3 h, and the heating rate was 5 °C/min. The activated products were purified with 1 M of HCl solution and deionized water and then dried in a vacuum at 110 °C for 24 h. The obtained samples were named CAC_T-R_.

### 2.3. Characterization

The crystal structure was studied by an XRD-700S/L X-ray diffractometer (XRD, Shimadzu, Kyoto, Japan). Raman measurements were performed using a Renishaw inVia Raman spectrometer with a 532 nm wavelength laser source (Raman, Renishaw, London, UK). Fourier transform infrared spectroscopy was carried out by a Nicolet iS50 FTIR spectrophotometer (FTIR, Thermo Fisher Scientific, Waltham, MA, USA). The morphology and structure were analyzed using a QUANTA250 field emission scanning electron microscope (SEM, FEI, Waltham, MA, USA). N_2_ adsorption–desorption isotherms were performed via an ASAP2460 analyzer (Micromeritics, Atlanta, GA, USA).

The detailed procedure of electrochemical measurements can be found in Supporting Information.

## 3. Results and Discussion

### 3.1. Structural Characterization

**Figure 1:**
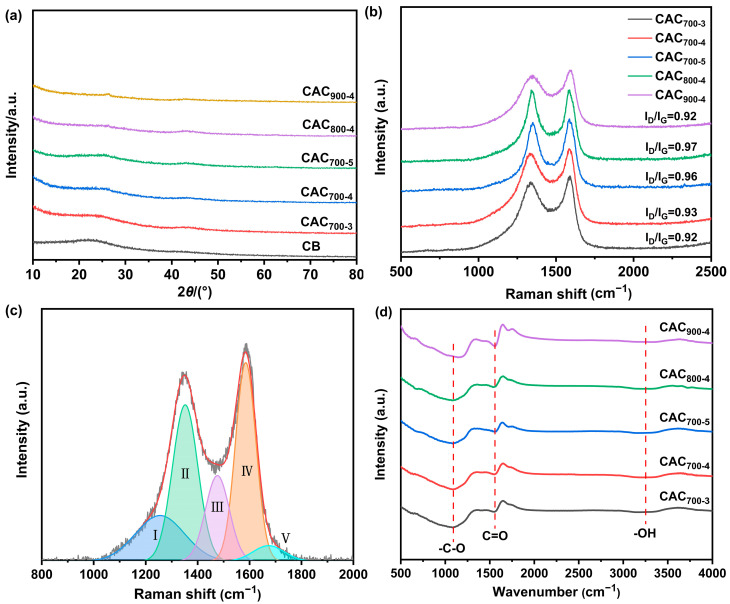
XRD patterns (**a**) and Raman spectra (**b**) of CB and CAC_T-R_ samples; representative Raman spectrum of CAC_700-4_ (**c**); and FTIR spectra of CAC_T-R_ (**d**).

The crystal structure of CB CAC_T-R_ activated carbon samples was analyzed by XRD spectroscopy. As shown in Figure 1, all the samples presented two diffraction peaks at 2θ values of 25° and 43°, which were attributed to the (002) and (100) crystal planes of the graphitized carbon, respectively [20]. It was found that the peaks get broader than that of CB. The two diffraction peaks show broadened peak shapes and weak peak intensities, indicating that CAC_T-R_ samples are dominated by amorphous carbon. In addition, CAC_900-4_ presented a relatively sharp peak at 2θ values of 25°, indicating that a higher activation temperature can enhance the graphitizing degree of the activated carbon. The structure of the CAC_T-R_ samples was further investigated by Raman spectroscopy. As shown in Figure 1b, all samples showed typical characteristic peaks of carbon materials at ~1350 and 1585 cm^−1^. The former peak (D band) was related to the defect and degree of disorder of the carbon materials. The latter peak (G band) was related to the in-plane vibration of sp2-hybridized carbon atoms. As presented in previous work, the Raman spectra were fitted using the Gaussian model, as presented in Figure 1c [21]. The shoulder peak Ⅰ is associated with the vibrations of aromatic rings. Peak Ⅱ (D band) is related to the breathing vibrations of non-perfect graphitic aromatic structures. Peak Ⅲ is related to the non-hexagonal rings. Peak Ⅳ (G band) is related to the in-plane vibration of sp2-hybridized carbon atoms. Peak Ⅴ appears due to the presence of carbonyl groups in the samples. Thus, the I_D_/I_G_ values calculated from the ratio between the areas of peak Ⅱ and peak Ⅳ were used to estimate the graphitization or defect degree of the carbon samples. The calculated I_D_/I_G_ values of CAC_700-3_, CAC_700-4_, CAC_700-5_, CAC_800-4,_ and CAC_900-4_ are 0.92, 0.93, 0.96, 0.97, and 0.92, respectively. A lower I_D_/I_G_ value indicates that CAC_900-4_ is more graphitized. Figure 1d shows the FTIR spectra of CAC_T-R_. The broad peak at 1000–1200 cm^−1^ corresponds to -CO stretching vibration. The peak centered at 1570 cm^−1^ was caused by C=O stretching vibration, and the peak at 3200–3500 cm^−1^ was due to the presence of -OH. The presence of oxygen-containing functional groups might improve the wettability of the electrode, as well as the energy storage ability of the electrode [22].

**Figure 2:**
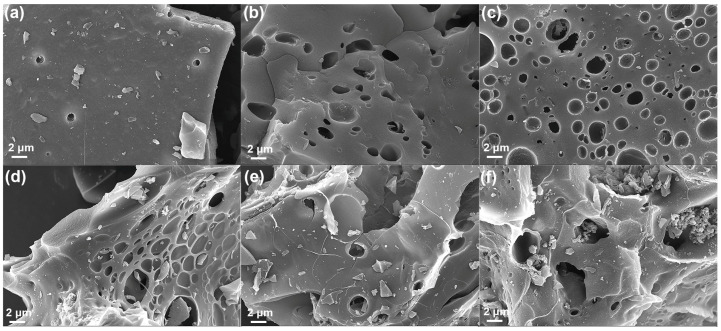
SEM images of (**a**) CB, (**b**) CAC_700-3_, (**c**) CAC_700-4_, (**d**) CAC_700-5_, (**e**) CAC_800-4_, and (**f**) CAC_900-4_.

SEM measurement was used to analyze the effect of alkaline/biochar ratios and activation temperature on the morphology and microstructure of the CB and CAC_T-R_ samples, as shown in Figure 2. The unactivated biochar CB exhibits a relatively smooth surface with a small number of holes (Figure 2a). The holes resulted from the volatilization of volatile fractions during the carbonization process. After KOH activation, plenty of holes appeared, which was attributed to the KOH etching of biochar (Figure 2b–d). It was also found that, as the KOH/biochar ratio increases, the surface of the activated carbons gradually becomes rougher and the number of holes increases. The sample CAC_700-4_ presented a dense porous structure. The porous structure should facilitate ion transport and electron transfer. This is attributed to the reaction between KOH and corncob carbon, which produces etching holes. However, the sample CAC_700-5_ presents a relatively rough structure with large pores, which was due to excessive KOH corrosion. It can be found that CAC_700-4_ retains an integrated structure with numerous pores. However, when the activation temperature exceeded 700 °C, the CAC_800-4_ and CAC_900-4_ were strongly affected by KOH activation, resulting in a rough surface with a collapsed tunnel structure. That was because, at an activation temperature of 700 °C, the activation process is mainly focused on pore formation and pore expansion, resulting in a dense porous structure. When the activation temperatures exceeded 700 °C, the inter-connected channel was over-etched, leading to structural collapse.

**Figure 3:**
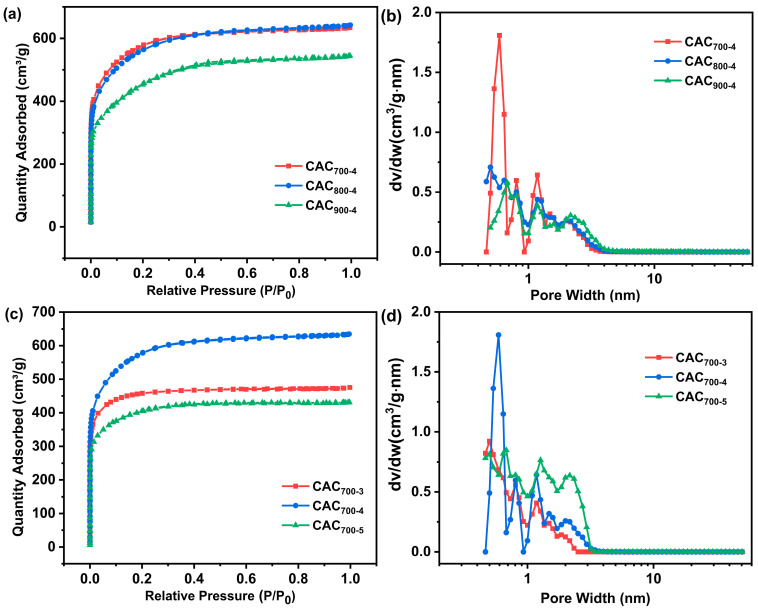
N_2_ adsorption–desorption isotherms (**a**,**c**) and pore size distribution curves (**b**,**d**) of CAC_T-R_ samples.

N_2_ adsorption-desorption isotherms were carried out to investigate the microstructure of the CAC_T-R_ samples. As shown in Figure 3a,c, CAC_T-R_ displayed a combined I/IV type adsorption–desorption isotherm. Rapid rising adsorption in the range of P/P_0_ < 0.1 indicates the presence of abundant micropores, and the hysteresis loop in the range of P/P_0_ > 0.4 indicates the presence of mesopores. The surface areas of LAC_T-R_ were calculated via the Brunauer–Emmett–Teller (BET) model, and the pore size distribution curves were obtained via the DFT model. As shown in Figure 3b,d, the samples have hierarchical porous structures with micropores and mesopores, which are consistent with the results of the N_2_ adsorption–desorption isotherms. The BET surface areas and pore volume parameters of the CAC_T-R_ samples are summarized in Table 1. The activation temperature had an apparent effect on the porous structure of the samples. As illustrated in Table 1, the surface area decreased with increasing activation temperature. The proportion of micropores decreases with increasing activation temperature, while the proportion of mesopores increases. This is because when the activation temperature reaches 900 °C, the carbon skeleton is over-etched by KOH. The KOH/biochar ratio also had an apparent effect on the porous structure of samples. At a KOH/biochar ratio of 3:1, the sample presented a relatively low surface area and pore volume due to inefficient KOH etching. As the KOH/biochar ratio increased to 4:1, a considerable number of micropores were produced due to the efficient activation of KOH. Thereby, the sample CAC_700-4_ gives a high surface area and pore volume. However, excessive dosage of KOH might cause over-etching of the carbon skeleton, leading to the collapsed structure of CAC_700-5_. These results are consistent with the above SEM analysis.

### 3.2. Electrochemical Performance Testing

**Figure 4:**
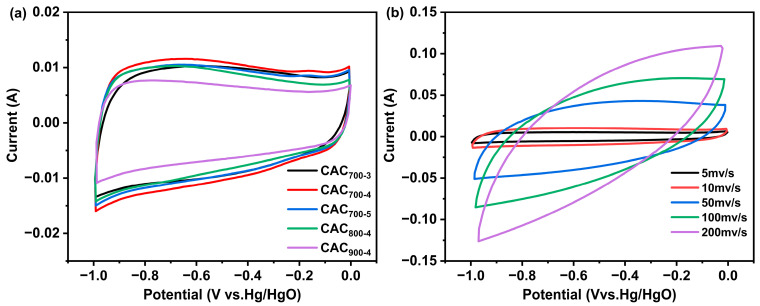
Cyclic voltammograms of CAC_T-R_ at 10 mV/s in 6 M KOH (**a**) and cyclic voltammograms of CAC_700-4_ at different scan rates (**b**).

As presented in Figure 4a, the CV curves of CAC_T-R_ samples exhibit near rectangular shapes, confirming their double-layer capacitance behavior [23]. The sample CAC_700-4_ exhibited the highest integral area, implying the optimal specific capacitance. The capacitive performance of CAC_700-4_ was further investigated by cyclic voltammograms under different scan rates (Figure 4b). As the scanning speed increased, the shape of cyclic voltammograms deviated from the ideal rectangular shape. This is primarily due to the fact that the sample CAC_700-4_ was predominantly composed of microporous structures. As the scanning rates increased, insufficient time hindered the diffusion and adsorption of electrolyte ions, thereby preventing the formation of a double electric layer [24].

**Figure 5:**
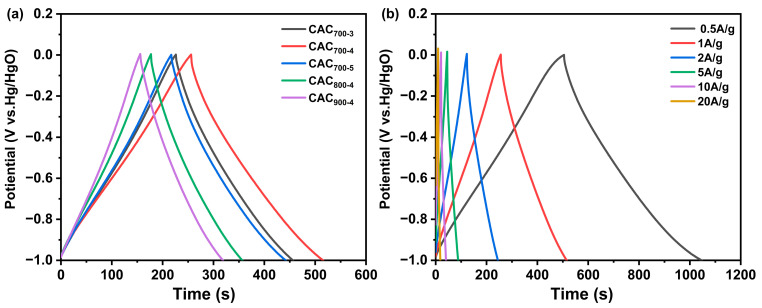
Galvanostatic charge–discharge curves of CAC_T-R_ at 1 A/g (**a**) and galvanostatic charge–discharge curves of CAC_700-4_ at different current densities (**b**).

As shown in Figure 5, the discharge curves of CAC_T-R_ samples exhibit a typical triangle shape, indicating that the samples possess excellent double-layer capacitance. The specific capacitance of CAC_700-3_, CAC_700-4_, CAC_700-5_, CAC_800-4_, and CAC_900-4_ were 231, 260, 226, 180, and 163 F/g, respectively. Among the samples, CAC_700-4_ exhibited the best capacitance performance, which is higher or comparable with that of other corncob-based carbon materials [18,21,25,26]. It is known that micropores mainly contribute to the storage of charge and ion transport, and mesopores can facilitate ion transport. In this case, the best capacitive performance of CAC_700-4_ can be attributed to its high surface area and high V_micro_. As illustrated in Figure 5b, with an increasing current density, the triangles become narrower and more acute; thus, the specific capacitance decreases. When the current density is 0.5 A/g, the specific capacitance of the CAC_700-4_ electrode was 271 F/g, and when the current density increased to 20.0 A/g, the specific capacitance was maintained at 70%.

**Figure 6:**
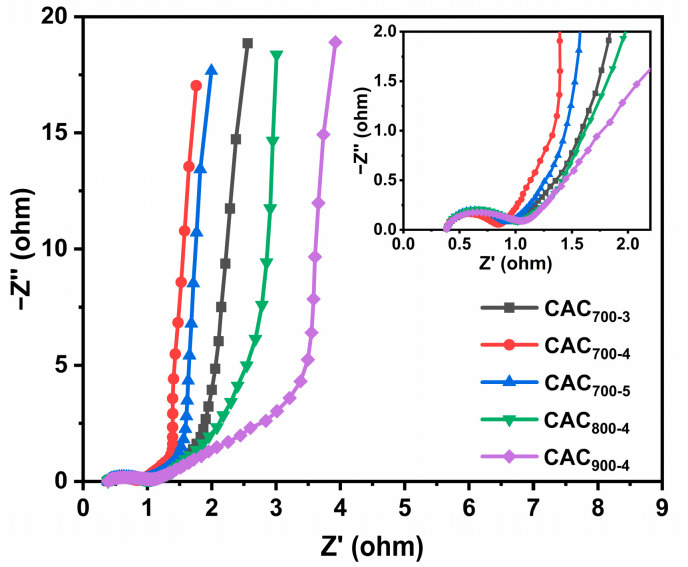
Nyquist plots of CAC_T-R_ samples.

The electrochemical impedance performance of the CAC_T-R_ samples was analyzed by Nyquist plots, as shown in Figure 6. The intercept of Nyquist plots with the *x*-axis corresponds to the equivalent series resistance (R_s_). The R_s_ values of CAC_T-R_ were all less than 0.5 Ω, indicating that electrolyte resistance and the electrode inherent resistance are low. The radius of the half-circle represents the interfacial charge–transfer resistance (R_ct_) [27]. A smaller radius indicates a faster charge transfer. The R_ct_ values of CAC_x_ recorded are in the order of CAC_700-4_ < CAC_700-5_ < CAC_700-3_ < CAC_800-4_ < CAC_900-4_. The low R_ct_ value (0.52 Ω) of CAC_700-4_ implies the rapid charge transfer during the electrochemical process, which benefits from the favorable distribution of micro- and mesopores in CAC_700-4_.

**Figure 7:**
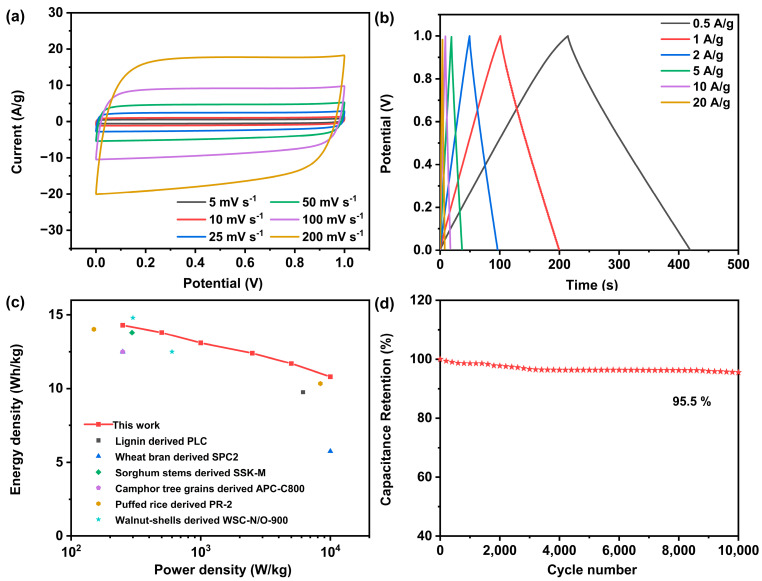
Electrochemical performances of CAC_700-4_//CAC_700-4_ symmetric supercapacitor using 6 M KOH as the electrolyte. (**a**) CV curves at scan rate of 10–200 mV/s. (**b**) GCD curves at current densities of 0.5–20 A/g. (**c**) Ragone plots and comparison with other reported results. (**d**) Cycling stability over 10,000 cycles at 10 A/g.

In order to evaluate the practical applicability of the prepared carbon materials, a two-electrode symmetrical supercapacitor (CAC_700-4_//CAC_700-4_) was fabricated and tested. Figure 7a shows the CV curves of the CAC_700-4_//CAC_700-4_ symmetric supercapacitor at different scan rates. The quasi-rectangular shape of CVs exhibited no obvious deformation as the scanning rate increased, suggesting its excellent capacitance response and reversibility. As shown in Figure 7b, the GCD curve still presented a symmetrical triangle shape at 20 A/g, indicating remarkable electrochemical reversibility and the rapid transport of electrolyte ions in the CAC_700-4_//CAC_700-4_ symmetric supercapacitor. When the charge–discharge current density was 0.5 A/g, the specific capacitance was 205.4 F/g. The supercapacitor showed a high capacitance retention of 75.9%, as the specific capacitance still reached 156 F/g when the current density increased to 20 A/g. The energy and power densities of the CAC_700-4_//CAC_700-4_ symmetric supercapacitor were calculated and presented by Ragone plots (Figure 7c). The CAC_700-4_//CAC_700-4_ symmetric supercapacitor exhibited an energy density of 14.3 Wh/kg at a power density of 250 W/kg, which is higher or comparable with that of some other biomass-derived carbon-based symmetric supercapacitors [28,29,30,31,32,33]. The energy density was maintained at 10.8 Wh/kg at the high-power density of 10,000 W/kg. Furthermore, the CAC_700-4_//CAC_700-4_ symmetric supercapacitor exhibited high cycling stability with a capacitance retention of 95.5% after 10,000 cycles at a current density of 10 A/g.

## 4. Conclusions

In this work, a series of corncob-derived activated carbons were prepared using traditional carbonization and KOH activation methods for high-performance supercapacitors. The alkaline/biochar ratio and activation temperature had a remarkable influence on the morphology, pore size distribution, and capacitance performance of as-prepared activated carbons. The samples presented a porous structure with a large number of micropores ranging from 0.5 to 2 nm and a small number of mesopores. The micropores contribute to the charge storage, while the mesopores benefit the transfer of ions. The optimal sample CAC_700-4_ exhibited the highest specific capacitance of 260 F/g at 1 A/g. The assembled CAC_700-4_//CAC_700-4_ symmetric supercapacitor displayed a good capacitive performance with a capacitance of 205.4 F/g at 0.5 A/g and an energy density of 14.3 Wh/kg at 250 W/kg. The symmetric supercapacitor also displayed a high capacitance retention of 95.5% after 10,000 cycles. The remarkable capacitance performance can be attributed to the high effective surface area and reasonable pore size distribution. This work provided the possibility of the high-value utilization of biomass waste in high-performance supercapacitors and can aid in alleviating environmental pollution.

## Figures and Tables

**Table 1:** Pore textural properties of CAC_T-R_ samples.

Samples	S_BET_^a^	V_total_^b^	V_micro_^c^	V_micro_/V_total_	D_ave_ (nm)
	(m^2^/g)	(m^3^/g)	(m^3^/g)		
CAC_700-4_	1945.70	0.9812	0.9122	92.96%	2.0173
CAC_800-4_	1919.16	0.9932	0.8965	90.26%	2.0701
CAC_900-4_	1627.58	0.7534	0.5002	66.39%	2.3861
CAC_700-3_	1482.41	0.7347	0.6971	94.88%	1.9825
CAC_700-5_	1470.09	0.6671	0.3785	56.73%	2.3912

^a^ Surface area calculated via the BET method. ^b^ Total pore volume measured at P/P_0_ = 0.99. ^c^ Micropore volume determined using the t-plot method.

## Data Availability

The original contributions presented in the study are included in the article/Supplementary Material, further inquiries can be directed to the corresponding authors.

## Supplementary Materials

The following supporting information can be downloaded at: https://www.mdpi.com/article/10.3390/ma17174341/s1, Supporting Information: Electrochemical measurements.

## References

[1] Bhat M.Y., Hashmi S.A., Khan M., Choi D., Qurashi A. (2023). Frontiers and recent developments on supercapacitor’s materials, design, and applications: Transport and power system applications. J. Energy Storage.

[2] Manasa P., Sambasivam S., Ran F. (2022). Recent progress on biomass waste derived activated carbon electrode materials for supercapacitors applications-A review. J. Energy Storage.

[3] Yin J., Zhang W.L., Alhebshi N.A., Salah N., Alshareef H.N. (2020). Synthesis strategies of porous carbon for supercapacitor applications. Small Methods.

[4] Wang R., Jayakumar A., Xu C., Lee J.M. (2016). Ni(OH)_2_ nanoflowers/graphene hydrogels: A new assembly for supercapacitors. ACS Sustain. Chem. Eng..

[5] Sun T., Li Z., Liu X., Ma L., Wang J., Yang S. (2016). Facile construction of 3D graphene/MoS_2_ composites as advanced electrode materials for supercapacitors. J. Power Sources.

[6] Czagany M., Hompoth S., Keshri A.K., Pandit N., Galambos I., Gacsi Z., Baumli P. (2024). Supercapacitors: An efficient way for energy storage application. Materials.

[7] Li H.L., Cao L.H., Zhang H.J., Tian Z.W., Zhang Q., Yang F., Yang H.Q., He S.J., Jiang S.H. (2022). Intertwined carbon networks derived from Polyimide/Cellulose composite as porous electrode for symmetrical supercapacitor. J. Colloid Interface Sci..

[8] Baykara E.A., Zeybek B. (2023). The preparation of polypyrrole/carboxyl functionalized multi-walled carbon nanotube@cobalthexacyanoferrate hybrid composite for high-performance supercapacitor application. J. Energy Storage.

[9] Hwang H.C., Woo J.S., Park S.Y. (2018). Flexible carbonized cellulose/single-walled carbon nanotube films with high conductivity. Carbohyd. Polym..

[10] Wang Y.Z., Liu Y.X., Wang D.H., Wang C., Guo L., Yi T.F. (2020). Free-standing honeycomb-like N doped carbon foam derived from coal tar pitch for high-performance supercapacitor. Appl. Surf. Sci..

[11] Yin Y., Liu Q., Zhao Y., Chen T., Wang J., Gui L., Lu C. (2023). Recent progress and future directions of biomass-derived hierarchical porous carbon: Designing, preparation, and supercapacitor applications. Energy Fuels.

[12] Zeng L., Thiruppathi A.R., Zalm J.v.d., Li X., Chen A. (2022). Biomass-derived amorphous carbon with localized active graphite defects for effective electrocatalytic N_2_ reduction. Appl. Surf. Sci..

[13] Yan D., Liu L., Wang X., Xu K., Zhong J. (2022). Biomass-derived activated carbon nanoarchitectonics with hibiscus flowers for high-performance supercapacitor electrode applications. Chem. Eng. Technol..

[14] Laheäär A., Przygocki P., Abbas Q., Béguin F. (2015). Appropriate methods for evaluating the efficiency and capacitive behavior of different types of supercapacitors. Electrochem. Commun..

[15] Jin T., Su J., Luo Q., Zhu W., Lai H., Huang D., Wang C. (2022). Preparation of N, P co-doped porous carbon derived from daylily for supercapacitor applications. ACS Omega.

[16] Wang Y., Li H., Yang W., Jian S., Zhang C., Duan G. (2022). One step activation by ammonium chloride toward N-doped porous carbon from camellia oleifera for supercapacitor with high specific capacitance and rate capability. Diam. Relat. Mater..

[17] Arkhipova E.A., Novotortsev R.Y., Ivanov A.S., Maslakov K.I., Savilov S.V. (2022). Rice husk-derived activated carbon electrode in redox-active electrolyte—New approach for enhancing supercapacitor performance. J. Energy Storage.

[18] Zhang Y., Zhao Y.P., Qiu L.L., Xiao J., Wu F.P., Cao J.P., Bai Y.H., Liu F.J. (2022). Insights into the KOH activation parameters in the preparation of corncob-based microporous carbon for high-performance supercapacitors. Diam. Relat. Mater..

[19] Shrestha L.K., Shahi S., Gnawali C.L., Adhikari M.P., Rajbhandari R., Pokharel B.P., Ma R., Shrestha R.G., Ariga K. (2022). *Phyllanthus emblica* seed-derived hierarchically porous carbon materials for high-performance supercapacitor applications. Materials.

[20] Merin P., Jimmy Joy P., Muralidharan M.N., Veena Gopalan E., Seema A. (2021). Biomass-derived activated carbon for high-performance supercapacitor electrode applications. Chem. Eng. Technol..

[21] Ortiz-Olivares R.D., Lobato-Peralta D.R., Arias D.M., Okolie J.A., Cuentas-Gallegos A.K., Sebastian P.J., Mayer A.R., Okoye P.U. (2022). Production of nanoarchitectonics corncob activated carbon as electrode material for enhanced supercapacitor performance. J. Energy Storage.

[22] Lobato-Peralta D.R., Arias D.M., Okoye P.U. (2021). Polymer superabsorbent from disposable diaper as a sustainable precursor for the development of stable supercapacitor electrode. J. Energy Storage.

[23] Shi F.Y., Tong Y., Li H.S., Li J.J., Cong Z.Y., Zhai S.R., An Q.D., Wang K. (2022). Synthesis of oxygen/nitrogen/sulfur codoped hierarchical porous carbon from enzymatically hydrolyzed lignin for high-performance supercapacitors. J. Energy Storage.

[24] Wang T., Liu Z.G., Li P.F., Wei H.Q., Wei K.X., Chen X.R. (2023). Lignin-derived carbon aerogels with high surface area for supercapacitor applications. Chem. Eng. J..

[25] Genovese M., Jiang J.H., Lian K., Holm N. (2015). High capacitive performance of exfoliated biochar nanosheets from biomass waste corn cob. J. Mater. Chem. A.

[26] Yang S.R., Zhang K.L. (2018). Converting corncob to activated porous carbon for supercapacitor application. Nanomaterials.

[27] Wang H., Xiong F.Q., Yang J.M., Ma B.L., Qing Y., Chu F.X., Wu Y.Q. (2022). Preparation of size-controlled all-lignin based carbon nanospheres and their electrochemical performance in supercapacitor. Ind. Crop. Prod..

[28] Fu F., Yang D., Zhang W., Wang H., Qiu X. (2020). Green self-assembly synthesis of porous lignin-derived carbon quasi-nanosheets for high-performance supercapacitors. Chem. Eng. J..

[29] Zhang L., Wang Y.Y., Yang S.W., Zhao G.Z., Han L., Li Y.J., Zhu G. (2022). Biomass-derived S, P, Cl tri-doped porous carbon for high-performance supercapacitor. Diam. Relat. Mater..

[30] Feng T., Wang S., Hua Y., Zhou P., Liu G., Ji K., Lin Z., Shi S., Jiang X., Zhang R. (2021). Synthesis of biomass-derived N, O-codoped hierarchical porous carbon with large surface area for high-performance supercapacitor. J. Energy Storage.

[31] Hao J.Y., Wang B.X., Xu H., Du J.C., Wu C., Qin W., Wu X.Q. (2024). Interfacial regulation of biomass-derived carbon towards high-performance supercapacitor. J. Energy Storage.

[32] Yuan Y.D., Sun Y., Liu C.G., Yang L., Zhao C.Z. (2023). Influence of KHCO_3_ Activation on characteristics of biomass-derived carbons for supercapacitor. Coatings.

[33] Liu Z.T., Li L.S., Wang M.L., Wu F.Z., Jin H.X., Wang Y. (2024). 3D hierarchical porous N, O co-doped carbon derived from the waste walnut shell via one-step carbonization for high-performance supercapacitor. Electroanalysis.

